# Musculoskeletal Injury in Australian Infantry Personnel: A Cross-sectional Study to Understand Prevention Priorities

**DOI:** 10.1093/milmed/usae427

**Published:** 2024-09-19

**Authors:** Joanne Stannard, Caroline Finch, Paula Dabovich, Lauren Fortington

**Affiliations:** School of Medical and Health Sciences, Edith Cowan University, Perth, WA 6027, Australia; School of Medical and Health Sciences, Edith Cowan University, Perth, WA 6027, Australia; School of Public Health, The University of Adelaide, Adelaide, SA 5005, Australia; School of Medical and Health Sciences, Edith Cowan University, Perth, WA 6027, Australia

## Abstract

**Introduction:**

Musculoskeletal injury patterns are under-investigated in the Royal Australian Infantry Corps. Subsequently, more evidence is needed to support injury prevention processes in this population. One difficulty in collecting injury information to monitor injury patterns within combat populations accurately is known injury concealment behaviors in such populations. This study aims to examine musculoskeletal injury epidemiology within Australian infantry battalions using a tailored approach to mitigate reporting avoidance.

**Materials and Methods:**

A cross-sectional study using an anonymous online survey captured musculoskeletal injury information directly from personnel serving within 2 Australian infantry battalions. The survey requested information on participants’ injury frequency in the previous 12 months and the context of participants’ most severe injury. Injury context was restricted to the most severe during the period to limit recall bias. The applied injury case definition encompassed all injuries that affected an individual’s ability to perform in their role. A descriptive analysis of all data recorded across the 2 battalions was conducted. Subgroup statistical difference was assessed by examining the 95% CI overlap between groups. The Department of Defence and Veterans’ Affairs Human Research Ethics Committee granted ethical approval for this study.

**Results:**

Overall, 166 individuals self-reported at least 1 injury in the past 12 months, representing a period prevalence of 55.5% (95% CI, 49.8-61.0%). No statistically significant prevalence differences existed between employment type, age, or sex. Approximately a quarter of injured participants were medically reclassified because of their injury, impacting their deployment fitness (*n* = 40, 24.4%). The following results relate to the most severe injury personnel experienced. Most injuries were service-related (*n* = 152, 91.6%). Field activities (*n* = 64, 39.3%) and physical training (*n* = 59, 36.2%) were the most common injury-related activities. Running was the most reported injury mechanism (*n* = 35, 21.7%), followed by pack marching (*n* = 29, 18.9%) and fall, slip, or trip (*n* = 18, 11.2%).

**Conclusion:**

Musculoskeletal injuries are common in the Australian infantry and significantly burden the workforce. Physical training and field exercises are most associated with injury and represent opportunities for injury risk-mitigation strategies to support the overall deployability of personnel and the combat effectiveness of their battalions. Future research should more formally explore the injury risk factors related to these activities using more robust study designs to collect injury and exposure information more accurately and reliably. One study strength includes using military-specific international injury surveillance guidelines to inform the survey design, to collect the recommended injury information for effective surveillance, and to enable future research comparison. A second study strength was tailoring the survey to promote participatory engagement, providing a high completion rate. A challenge in conducting this research was coordinating participant recruitment and data collection during domestic operations. Such challenges reflect the reality of conducting research in the military.

## INTRODUCTION

Infantry soldiers support military missions by serving as the ground combat force to deter enemy forces.^1^ The Australian Army’s basic military and infantry training takes approximately 6 months to complete. Australian infanteers continue their physical and military skills training within their battalions to ensure operational fitness and deployment readiness. The role of an infanteer is physically arduous, including prolonged load carriage and mobilizing heavy weaponry.^1,2^ Infantry personnel are exposed to additional occupational stressors, often simultaneously, such as rugged terrain, sleep deprivation, and austere environments. Subsequently, infantry personnel are at risk of being substantially burdened by musculoskeletal injury.^3^ Preventing injuries in the Australian infantry is essential to protect personnel and preserve combat capability.

Military training and service are associated with musculoskeletal injury.^3–7^ International research reports that approximately half of infantry personnel sustain an injury within 1 year, adversely affecting their performance.^3,7^ Understanding the number, nature, and cause of injury through surveillance is essential to its prevention.^8,9^ Injury surveillance is a foundational step in preventative health frameworks where epidemiological information informs the design and evaluation of interventions to mitigate injury.^8,9^ Presently, there is a void in research investigating musculoskeletal injury epidemiology in the Australian infantry. Subsequently, little evidence exists to direct prevention efforts that may otherwise help preserve individual and military capability.

Further to limited epidemiological information, international research has highlighted that many combat personnel avoid seeking medical help when injured for various reasons, such as avoiding potential career consequences.^10–16^ Injury concealment behaviors can complicate accurate monitoring of injury patterns, as current surveillance methods rely on personnel engaging with the health system to collect data. Subsequently, health care surveillance systems that monitor injuries in military settings will likely underestimate injury prevalence. Active surveillance, a process of collecting injury information directly from individuals and tailored to the population, is warranted to help mitigate reporting avoidance and support accurate insights into this population.^17^

This study describes the epidemiology of musculoskeletal injury in Australian Army infantry personnel. Data collection was tailored to alleviate potential injury reporting concerns and support open reporting by personnel.

## METHODS

A cross-sectional study was conducted using an online survey to collect descriptive injury epidemiology data directly from personnel of 2 infantry battalions. The Department of Defence and Veterans’ Affairs Human Research Ethics Committee granted ethical approval (protocol 416-22). This study’s reporting methods align with the STROBE statement for cross-sectional studies.^18^

### Recruitment

Participants were recruited from 2 infantry battalions of a single brigade between June and July 2022. All ranks and those serving full-time, including support staff, were eligible to partake. A recruitment email was delivered to battalion members the week preceding data collection. Potential participants available on the survey data collection day also received a face-to-face informative brief to encourage trust with the research team. The email and brief emphasized that participants could partake in the study whether or not they sustained an injury in the previous 12 months. The command team was not used to recruit participants to avoid coercion and mitigate real or perceived power imbalances. Individual consent was obtained before survey access was provided. Participants were excluded if they were under the age of 18 years or serving as an Army reservist in a part-time capacity. To determine injury prevalence accuracy across the 2 battalions, at least 285 participants were needed. This number was based on a satisfactory margin of error (5%) within the 2 battalions 1100-person population.

### Injury Case Definition

The term “musculoskeletal” was explained to participants at the beginning of the questionnaire as the human body’s muscles, skeleton, joints, tendons, and ligaments. The injury case definition was a musculoskeletal injury that impacted an individual’s ability to perform in their role in the past 12 months. This case definition was designed to minimize recall bias (by limiting the recall period), focus on a metric of interest to commanders, such as injuries that impact performance, and exclude minor ailments that could compromise recall accuracy.^19,20^

### Questionnaire Design

Questions predominately prompted closed-option responses. Forced response functions were used to minimize missing data. Display logic functions were used to ensure that only relevant questions were presented to participants for survey efficiency; for example, only respondents who indicated they sustained an injury were asked for further details about their injury. The questionnaire was built with online software (Qualtrics) to be smartphone-friendly and permit survey access via a QR code. Responses were anonymous to protect participants’ privacy and minimize injury reporting barriers known in this population, such as reluctance to provide accurate accounts of personal injury.

Up to 35 questions were presented to participants, depending on participant choices and subsequent display logic. All data items and their categorization methods can be viewed in Supplementary Material 1. Questions were adapted from military-specific injury surveillance guidelines^17^ and the World Health Organization’s injury surveillance recommendations.^21^ Activity and injury mechanism categories were created because of the absence of suitable guidelines for recording military-specific activity and injury mechanisms. A serving infantry officer not associated with the study reviewed the categories designed to ensure they were consistent with infantry activities. The same infantry officer also assessed the survey language to help minimize the use of technical or medical jargon and ensure participants would easily understand the questions.

Initial survey questions screened participants for eligibility. The survey progressed to musculoskeletal injury-related questions requesting information for all injuries sustained during the preceding 12-month period that impacted their work performance. The questionnaire progressed to requesting information about injury circumstances relating to what participants considered their most severe injury in the study period if a participant indicated they had sustained an injury. Past research suggests that severe injuries are associated with improved recall accuracy relative to minor ailments.^19^ Thus, restricting contextual questions to participants’ single most severe injury was a control measure to improve data accuracy.

### Data Analysis

A descriptive analysis was conducted, reporting all data recorded collectively across the 2 battalions. Musculoskeletal injury counts, proportions, and period prevalence were computed. Poisson 95% CIs were calculated using statistical software for all proportions. Descriptive analysis was conducted to compare subgroup injury prevalence proportions for age brackets and sex groups, as well as commissioned officers with enlisted soldiers and infantry and non-infantry roles. Subgroup difference was assessed by examining the 95% CI overlap between groups. If confidence interval overlap occurred, the difference between the 2 groups was considered statistically insignificant. A statistical significance was deemed found in the instance of no confidence interval overlap. Data suppression techniques were used to preserve individual privacy when the total count for a data item was less than 5, for example, by integrating similar data items to form 1 group with a count equal to or greater than 5. Respondent dropout was accounted for by adjusting the denominators for each item based on valid responses only. The total respondent numbers for data items are presented in the results tables.

## RESULTS

In total, 306 participants commenced the questionnaire, giving an overall response rate from the 2 battalions of 27.8%. Seven individuals did not meet the eligibility criteria, leaving 299 eligible participants. Overall, 296 surveys were completed in full, giving a 96.7% survey completion rate. No reason was ascertained for non-participation or participant attrition at any stage. The median survey completion time was 3.5 min. The median participant age was 23 years (range 18–65 years) (**Table 1**). Most participants were male (sex: *n* = 287, 96.0%), in an infantry role (*n* = 257, 86%) and serving as enlisted soldiers (*n* = 280, 93.6%).

**Table 1.** Participant Demographics Across the 2 Infantry Battalions

	All respondents			Injured respondents		
Demographics	n	%	95% CI	*n*	%	95% CI
Total participants	299	–	–	166	55.5	49.8–61.0
Age in years, median (range)	23 (18–65)	–	–	24	–	–
18–22	–	–	–	64	52.0	43.3–60.7
23–27	–	–	–	62	55.9	46.6–64.7
28–32	–	–	–	26	71.0	52.5–80.9
33+	–	–	–	14	52.9	34.0–69.3
Sex^a^	–	–	–			
Male	287	96.0	93.1–97.7	161	56.1	50.3–61.7
Female	12	4.0	2.3–6.9	5	41.7	32.0–80.7
Rank						
Soldiers	280	93.6	90.3–95.9	159	56.8	50.9–62.5
Commissioned officers	19	6.4	4.1–9.7	7	36.8	19.1–59.0
Army Corps						
Infantry	257	86.0	81.6–89.4	145	56.4	50.3–62.3
Other	42	14.0	10.6–18.4	21	50.0	35.5–64.5
Service duration						
Participants with >12 months	283	94.6	91.5–96.7	158	55.8	50.0–61.5
Participants with <12 months	16	5.4	3.3–8.5	8	50.0	28.0–72.0

a Sex and gender information was requested from participants, of which the distribution was the same.

There were 166 participants who self-reported at least 1 injury, representing a 55.5% (95% CI 49.8-61.0%) period prevalence in 12 months (**Table 1**). The number of separate injuries reported ranged from 1 to 7, cumulating to 303 musculoskeletal injuries. The injury prevalence by demographics identified some group differences; however, none were statistically significant. Proportionally, enlisted soldiers (56.8%, *n* = 159) reported more injuries than commissioned officers (38.9%, *n* = 7). Similar injury proportions were identified between infantry and non-infantry participants (infantry 56.4%, *n* = 145; non-infantry 50.0%, *n* = 21). Females reported fewer injuries than males (females 41.6%, *n* = 5; males 56.1%, *n* = 161).

The remaining results relate to the contextual information from the questions restricted to participants’ most severe musculoskeletal injury. Almost all injuries reported as participants’ most severe injury were service-related (*n* = 152, 91.6%) (**Table 2**). Most of these injuries occurred post-qualification training (70.6%), and approximately a third were sustained during qualification training (basic training *n* = 10, 6.1%; initial employment training *n* = 38, 23.3%). Most cases were new injuries (*n* = 120, 73.2%), and the remaining were past injury aggravations (*n* = 44, 26.8%). Of these injuries, most occurred suddenly, such as after a specific event or trauma (*n* = 94, 57.3%), with the remainder considered a more gradual onset (*n* = 70, 42.7%).

**Table 2.** The Number and Proportion of Injuries by Service-Related, Career Phase, Injury Onset, and Occupational Burden Based Upon Participants’ Most Severe Injury During the Study Period

Injury circumstances	Respondents (*n*)	Cases (*n*)	%	95% CI
Service-related injury	166	152	91.6	86.3–94.9
Injury history	164			
Primary injury		120	73.2	65.9–79.4
Aggravation of past injury		44	26.8	20.6–34.1
Career phase injury occurred	163			
Basic training		10	6.1	3.4–10.9
Initial employment training		38	23.3	17.5–30.4
After qualification training		115	70.6	63.1–77.0
Mode of injury onset	163			
Sudden onset		94	57.3	49.7–64.6
Gradual onset		70	42.7	35.4–50.3
Occupational impact of injury				
Number of days performance was impacted^a^	164			
Zero days		8	4.9	2.5–9.3
1 day to 1 week		28	17.1	12.1–23.6
1–2 weeks		17	10.4	6.6–16.0
2–4 weeks		30	18.3	13.1–24.9
>1 month		60	36.6	29.6–44.2
Permanently		21	12.8	8.5–18.8
Medical reclassification	164	40	24.4	18.5–31.5
Missed a military activity	164	64	39.0	31.9–46.7

a Overlap in time frames reflects the estimated time from recall.

Over one-third of participants reported that their injury affected their work performance for over 1 month (*n* = 60, 36.6%), and 12.8% (*n* = 21) felt permanently impaired (**Table 2**). Approximately a quarter of injured participants required their deployable medical status to change (*n* = 40, 24.4%). In the Australian Defence Force (ADF), personnel are permitted 28 temporary restricted duty days for health conditions without changing an individual’s medical suitability to deploy. A restriction requirement greater than this initiates a medical reclassification from deployable to deployable with permanent restricted duty or non-deployable. Thirty-nine percent (*n* = 64) of injured participants, or 21.4% of all respondents, reported missing a military activity because of injury, such as a training course or field exercise (**Table 2**).

Approximately half of the most severe injuries involved the lower limb (*n* = 85, 51.8%), followed by the trunk and spine (*n* = 39, 23.9%) and the upper limb (*n* = 26, 16.0%) (**Table 3**). The knee was the body part most affected (*n* = 34, 20.7%), followed by the lower back (*n* = 29, 17.7%).

**Table 3.** The Number and Proportion of Most Severe Injuries by Body Parts Affected (Respondents *n* = 164)

Body regions and parts	*n*	%	95% CI
Head/neck	13	8.0	4.7–13.1
Head, face, or jaw	5	3.0	1.3–6.9
Neck	8	4.9	2.5–9.3
Trunk/spine	39	23.9	17.9–30.8
Upper back, abdomen, or chest	10	6.1	3.3–10.9
Lower back	29	17.7	12.6–24.2
Upper limb	26	16.0	11.6–22.9
Shoulder	18	11.0	7.1–16.7
Arm	9	5.5	2.9–10.1
Lower limb	85	51.8	44.2–59.3
Hip, groin, or thigh	8	4.9	2.5–9.3
Knee	34	20.7	15.2–27.6
Lower leg	18	11.0	7.1–16.7
Ankle	18	11.0	7.1–16.7
Foot or toes	7	4.3	2.1–8.5

**Table 4** summarizes the injury circumstances for participants’ self-reported most severe musculoskeletal injury. Most injuries were sustained while participants were located on base (*n* = 70, 42.9%, or in field environments (*n* = 58, 35.6%). Almost all injuries occurred during the daytime (*n* = 150, 92.0%). Over one-third of injuries were sustained during combat or field-related activities (*n* = 64, 39.3%), followed by physical training (PT) (*n* = 59, 36.2%), while a substantial number of respondents were “not sure” (*n* = 15, 9.2%). Running was the most reported specific injury mechanism at the time of injury (*n* = 35, 21.7%), followed by pack marching (*n* = 29, 18.9%), and fall, slip, or trip (*n* = 18, 11.2%).

**Table 4.** The Context of Participants’ Self-Reported Most Severe Musculoskeletal Injury Occurred (Respondents *n* = 163)

Injury context	*n*	%	95% CI
Geographical location			
On base	70	42.9	35.6–50.6
Field environment	58	35.6	28.6–43.2
Non-work related site	8	4.9	2.5–9.4
Combat environment	5	3.1	1.3–7.0
Not sure	8	4.9	2.5–9.4
Other	14	8.6	5.2–13.9
Time of day			
Day	150	92.0	86.8–95.3
Night	13	8.0	4.7–13.2
Activity at the time of injury			
Combat/field	64	39.3	32.1–46.9
Physical training	59	36.2	29.2–43.8
Military skills training	12	7.4	4.3–12.4
Not sure	15	9.2	5.7–14.6
Other	13	8.0	4.7–13.2
Injury mechanism			
Running	35	21.7	16.1–28.7
Pack marching	29	18.9	12.8–24.7
Fall, slip, or trip	18	11.2	7.2–17.0
Lifting or moving heavy objects	18	11.2	7.2–17.0
Prolonged positioning	7	4.3	2.1–8.7
Jumping or landing from a height	6	3.7	1.7–7.9
Collision or tackle	5	3.1	1.3–7.1
Not sure	18	11.2	7.2–17.0
Other	25	15.5	10.7–21.9

## DISCUSSION

This study aimed to address a knowledge gap in the musculoskeletal injury epidemiology of Australian Army infantry personnel. Findings from this study indicate that musculoskeletal injury is a substantial occupational burden on individuals and collectively impacts the capability of the Australian infantry. Over half of the participants self-reported at least 1 injury impacting work performance in the previous year. Almost two-thirds of injured personnel reported their performance was affected for more than 1 month or permanently. One-fifth of the surveyed workforce reported missing a military activity because of injury. Among those injured, one-quarter experienced a change in their medical deployable status, representing 13.4% of the surveyed workforce. Concerning respondents’ most severe injury, nearly all were service-related. Military field exercises and PT were the most common injury-related activities, particularly when running or pack marching. These activities are recommended as priorities for future research using more robust study designs to investigate aetiology and trial interventions for injury prevention.

Over half of the respondents reported at least 1 injury in the previous 12 months that impacted their work performance. This injury prevalence is consistent with other international infantry populations.^3,7^ Although there was some injury prevalence variation across demographic groups, this was not statistically significant. In contrast to past research,^22–24^ this study suggests no significant injury difference between sex groups across the battalions. This finding must be interpreted cautiously as the study is limited in drawing an accurate conclusion between injury risk and sex because of the small number of female participants. All women participating were in non-combat roles, and therefore, combat roles and sex-related injury risk cannot be determined. Sex-related injury prevalence is essential to monitor because of the known injury risks in women in combat roles.^23,24^ As combat roles are increasingly more available for women to enter,^25,26^ along with the ADF recruitment targets to increase female representation in combat roles in the Australian Army,^27^ the ADF must monitor sex-related injury patterns as an essential step to ensuring the safe recruitment and retention of women in these roles.

The results of this study indicate no statistically significant difference in injury prevalence between non-combat and combat personnel. Existing infantry injury literature tends to investigate those in training compared to the trained workforce.^3,7,16,23,28,29^ Of the existing research in qualified infantry personnel, there are mixed findings comparing injury prevalence between combat and non-combat roles.^23,29^ Similar to research in the United Kingdom’s forces,^7,23^ this study found no statistically significant difference in injury prevalence between non-combat and combat personnel. Similar injury prevalence between these cohorts contradicts common anecdotal evidence within the military community of injury risk with employment types. The injury risk is typically considered higher in combat roles based on known exposure to more physically demanding duties. Similar injury prevalence in these cohorts may be attributed to several reasons. For example, lower physical fitness is a known injury risk factor in the military. In the ADF, combat personnel are held to a higher fitness standard at recruitment and during employment than non-combat personnel, which may be protective against injury.^30,31^ Thus, similar injury prevalence between combat and non-combat roles could be attributed to higher and protective fitness levels in infantry personnel despite more physically demanding duties. However, further research is needed to test such a hypothesis and would require the determination of confounding factors, such as pre-existing fitness levels and activity exposure time. Furthermore, this study’s limited number of non-combat personnel limits definitive conclusions. Future research investigating employment types’ injury risk should consider confounding variables and known risk factors, such as fitness and exposure to military activities when comparing risk between cohorts. Such information can help inform future recruitment and employment physical fitness standards policies.

Findings unique to this study relate to the greater contextual information exploring when and how the injury occurred, such as geographical place of occurrence, injury activities, and mechanism. Most injuries occurred while on a military base or in the field environment. Field activities were more commonly associated with injury than PT. This finding contrasts past international research, indicating that PT, sport, or running are the most common injury-related activities.^3,7^ One possible reason for this disparity could be the use of different injury surveillance methods and the restriction of our activity and injury mechanism data collection to a single and the most severe injury. Another reason could be the variance in the activities undertaken by infantry personnel and the time spent on these activities in other nations. Prospective study designs capturing exposure information are required to identify injury risks associated with these activities more accurately. Furthermore, consistent injury surveillance methods between nations are also recommended to compare injury outcomes between countries reliably.

Field exercises and PT were the most common activities associated with injury, including those of a severe nature requiring medical fitness reclassification. Although this study has provided preliminary evidence highlighting priorities for injury prevention in Australian infantry populations, higher quality evidence is required to adequately inform military organizations on prevention needs. Future research should investigate PT and field-related injury aetiology and injury risk factors using larger, more representative sample sizes and robust study designs, such as prospective studies that collect injury and exposure information. This information is essential to inform the subsequent steps recommended in preventative health frameworks,^8,9^ such as interventional trials. Only by applying this programmatic approach to injury prevention can we confidently inform military leaders and organizations of the injury prevention policy changes or programs necessary to mitigate injuries in infantry personnel.^9^ Furthermore, researchers must understand the context in which prevention strategies are to be introduced to assist in the practical translation of research.^8^ For example, it is unrealistic to mitigate injury risk entirely in the field or combat environment because of the inherent nature of war and training requirements. Military organizations accept this risk for national security reasons. Further investigations are needed to determine possible risk mitigation strategies that can be implemented practically and realistically in the field or combat environment.

There were several challenges in conducting this research. First, the study coincided with a domestic operation in response to unexpected natural disasters in the country; thus, arranging a suitable time with the battalions to maximize recruitment was difficult. Such challenges reflect the reality of conducting research in the military. This study surveyed 299 participants, meeting the 285 minimum sample requirements for calculating population prevalence; however, this participation number only represents one-quarter of personnel posted to the 2 battalions. Many personnel were inaccessible for recruitment, potentially influencing the participant pool. For example, some personnel who did not deploy in the domestic operations may have been medically unfit to do so. As such, the survey results may be impacted by selection bias because of the potential inclusion of a greater proportion of injured personnel who did not deploy. Another challenge was that on one day of the survey, a major mobile network provider was experiencing service issues, impacting some personnel completing the survey. Most incomplete surveys were recorded on this day; however, it is unknown if this also accounted for participant dropout.

Some study design limitations exist that may impact the interpretation of the study’s results. The results may be influenced by recall bias, a bias type where error is introduced when participants cannot accurately remember past events. Accordingly, the study’s methods were designed to minimize this risk by constraining information requests for the most severe injury and restricting the recall period to 12 months, which has been shown to improve recall in past research.^19,20^ Secondly, our recruitment processes had some limitations. Although not formally recorded, approximately 10 members who attended the information brief declined to participate. There is the potential that some personnel did not partake because they were reluctant to report injuries, or potentially only those previously impacted by injuries were motivated to participate. We were unable to collect information from non-respondents to confirm this. Lastly, these results reflect 2 infantry battalions from 1 brigade. Presently, there are 7 full-time infantry battalions within the Royal Australian Infantry Corps, and thus, the results may not accurately reflect the entire Corps.

One study strength includes using international injury surveillance guidelines in the survey design to collect the recommended information for effective surveillance and to facilitate future comparison with research using similar methods.^17,21^ A second strength of the study was that the survey was purposefully anonymous to promote participatory engagement, providing a high completion rate. The Australian Army could consider periodic anonymous self-report health surveys as part of active injury surveillance programs to proactively monitor injuries in populations associated with higher injury risk and where health care avoidance is known to occur, such as infantry populations.^16^ Alternatively, regular identifiable surveys to monitor injuries are standard practice in professional sports to identify emerging problems and initiate early intervention.^32,33^ Such survey techniques have recently been adopted in a military context.^34–36^ These injury monitoring strategies are well received by military personnel when rapport exists with supporting health staff.^36^ Future research is recommended to investigate health care avoidance in the Australian infantry and the strategies required to enhance health care engagement to improve early intervention and mitigate workplace presenteeism. Further strengths in this study include the use of several web-based survey strategies to increase response rates.^37^ For example, the survey was designed to take less than 10 min to complete to avoid respondent fatigue. The information brief and recruitment processes were face-to-face rather than emailed to increase engagement through rapport. Lastly, an online survey using QR codes allowed immediate and rapid survey distribution to minimize participant attrition.

## CONCLUSION

Based on the self-report accounts from Australian Army infantry personnel, musculoskeletal injuries are common in Australian infantry battalions and place adverse burdens on workforce productivity and military capability. Field exercises and PT are military activities most associated with injury and are recommended priority activities for future research to mitigate injuries in the Australian Army.

## Supplementary Material

usae427_Supp

## Data Availability

All data in support of the research findings are presented in this study. Further data from this study investigating a separate health topic are under embargo.

## CLINICAL TRIAL REGISTRATION

None declared.
