# Supplementary material for: Musculoskeletal Injury in Australian Infantry Personnel: A Cross-sectional Study to Understand Prevention Priorities
Source: Mil Med. 2024 Sep 19;190(3-4):e682–9. doi: 10.1093/milmed/usae427 (PMC11878795; doi:10.1093/milmed/usae427)
Supplement: usae427_Supp [file usae427_supp.zip › Military Medicine Submission - Supplementary Material 1.pdf]

Supplementary material 1: Self-report musculoskeletal injury survey

Thank you for taking part in this survey. We need to understand your injury history to recommend suitable solutions to reduce injuries in Australian infantry units in the future.

For this survey, we seek information about **musculoskeletal injuries only, meaning injuries related to your muscles, tendons, bones, and joints.**

The first few survey questions will request some information about yourself to help us understand the type of people within your unit.

Q1 I declare that I have signed a consent form and returned it to the research team.

Yes

No

Skip To: End of Block If Q1 = No

Q2 Are you currently a full-time member of the ADF or a reservist on continuous full-time service?

Yes

No

Skip To: End of Block If Q2 = No

Q3 Are you over the age of 18?

Yes

No

Skip To: End of Block If Q3 = No

Q4 How long have you been a full-time ADF member?

Less than 12 months

More than 12 months

Q5 How old were you on your last birthday?

18

19

20

21

22

23

24

25

26

27

28

29

30

31

32

33

34

59

60

61

62

63

64

65 plus

Q6 What was your sex recorded at birth?

Please note that a separate question on gender is also asked in the survey.

Male

Female

Another term (please specify) ____

Q7 How do you describe your gender?

Gender refers to current gender, which may be different to your sex recorded at birth and may be different to what is indicated on legal documents.

Man or male

Woman or female

Non-binary

I use a different term (please specify)____

Prefer not to say

Q8 What is your rank?

Other ranks (PTE)

Junior NCO (LCPL to CPL)

Senior NCO (SGT to WO1)

Commissioned officer

Q9 Which unit are you posted to?

5 RAR

7 RAR

Q10 What army corps do you belong to?

Royal Australian Infantry Corps

Royal Australian Armoured Corps

Royal Regiment of Australian Artillery

Royal Australian Engineers

Royal Australian Corps of Signals

Australian Army Aviation Corps

Australian Intelligence Corps

Royal Australian Corps of Transport

Royal Australian Army Medical Corps

Royal Australian Ordnance Corps

Royal Australian Electrical and Mechanical Engineers

Royal Australian Army Chaplains Department

Australian Army Legal Corps

Royal Australian Army Educational Corps

Royal Australian Corps of Military Police

Other ____

Display This Question:

If Q10 = Other

Q11 Please specify which army corps you belong to.

____

The following questions will help us understand how common injuries occur within your unit. Please answer the questions to the best of your ability.

Display This Question:

If Q4 = More than 12 months

Q12 Have you sustained any injuries within the past 12 months that impacted your ability to perform in your role?

Yes

No

Skip To: End of Survey If Q12 = No

Display This Question:

If Q4 = Less than 12 months

Q13 Have you sustained any injuries since being a full-time ADF member that impacted your ability to perform in your role?

Yes

No

Skip To: End of Survey If Q13 = No

Display This Question:

If Q12 = Yes

Q14 How many injuries did you sustain that impacted your ability to perform in your role in the last 12 months?

1

2

3

4

5

6

7

8

9

10

Greater than 10

Display This Question:

If Q13 = Yes

Q15 How many injuries did you sustain that impacted your ability to perform in your role since being a full-time ADF member?

1

2

3

4

5

6

7

8

9

10

Greater than 10

Q16 Of these injuries, how many did you seek help from a Joint Health Command health centre?

0

1

2

3

4

5

6

7

8

9

10

Greater than 10

Display This:

If Q12 = Yes

We would like you to tell us more about the most severe injury you sustained in the last 12 months for the remaining questions.

Display This:

If Q13 = Yes

We would like you to tell us more about the most severe injury you sustained since becoming a full-time ADF member for the remaining questions.

Q17 Did you seek help for this injury from a Joint Health Command health centre?

Yes

No

Display This Question:

If Q17 = No

Q18 Why did you not seek help from a Joint Health Command health centre?

Please select the answer which is most appropriate for your situation.

I did not think I needed medical assistance

I was concerned that my injury might affect future career opportunities

I wanted to avoid negative perceptions associated with injuries

I wanted to avoid being given a PM 101 (CHIT)

I have previously had a negative experience with medical providers

It was inconvenient to attend the health centre

Other (please specify) ____

Q19 Did the injury occur whilst on duty?

Yes

No

Display This Question:

If Q19 = Yes

Q20 Did you report this injury using Sentinel?

Yes

No

Display This Question:

If Q20 = No

Q21 Please describe the reasons why you did not report the injury using Sentinel.

____

Q22 Why did you consider this injury to be your most severe injury?
Please select one choice that suits your situation the most.

I only had one injury

It caused the most pain

It impacted my ability to work

I was unable to perform at my best

Other (please specify) ____

Q23 How many days in total did this injury impact your ability to perform in your role?

0 days

1 to 3 days

3 days to 1 week

1 to 2 weeks

2 to 4 weeks

Greater than 1 month

Permanently

Q24 Did this injury require you to be medically downgraded?

Yes

No

Q25 Did you miss any military activities due to this injury? For example, you could not attend a career course or participate in a field exercise.

Yes

No

Display This:

If Q12 = Yes

The following questions will help us understand the circumstance of this injury. We need this information to understand how we can prevent injuries in the future.

Remember, these questions are about the most severe musculoskeletal injury you sustained in the last 12 months.

Display This:

If Q13 = Yes

The following questions will help us understand the circumstance of this injury. We need this information to understand how we can prevent injuries in the future.

Remember, these questions are about the most severe injury you sustained since becoming a full-time ADF member.

Q26 Which body part was affected? If more than one body part was involved, please choose the most severely injured part.

Head

Face or jaw

Neck

Upper back

Lower back

Shoulder

Upper arm

Elbow

Forearm

Wrist

Hands or fingers

Chest

Abdomen

Hip or groin

Thigh

Knee

Lower leg

Ankle

Foot or toes

Q27 Was this injury an aggravation of a previous injury?

Yes

No

Q28 How did your injury or symptoms commence?

My symptoms occurred suddenly, e.g., your symptoms started immediately after a specific event or trauma

My symptoms happened gradually over time, e.g., your symptoms started slowly and progressively

Q29 At which phase of your career did this injury occur?

Basic Training Army Recruit Course (ARC) or Royal Military College (RMC)

Initial Employment Training (IET)

After qualification training

Q30 Which geographical location did the injury occur?

On base

At home

Non-work related site

Field environment

Combat environment

Waterbody or sea

Not sure

Other (please specify) ____

Q31 Did the injury occur during the day or night?

Day

Night

Q32 What activity were you doing at the time of injury?

PTI led PT

ADFFL led PT

Self-directed PT (e.g., your own gym or running program)

BFA

PESA

Military skills training

ADF organised sport

Non-ADF organised sport or recreational activities

Driving

Desk based job-related tasks

Combat / Field

Not sure

Other

Display This Question:

If Q32 = Military skills training

Q33 What military skill were you training at the time?

____

Display This Question:

If Q32 = ADF organised sport

Q34 Which sport were you playing at the time?

____

Display This Question:

If Q32 = Other

Or Q32 = Non-ADF organised sport or recreational activities

Q35 Please specify what activity you were doing at the time.

____

Q36 How did the injury occur?

Running

Fall, slip or trip

Pack marching

Kicking

Swimming

Throwing

Jumping or landing from a height

Lifting or moving heavy objects

Stepping or climbing

Side stepping, pivoting, changing direction or agility

Collision or tackle

Prolonged positioning (e.g., sitting or standing for an extended time)

Struck by an object

Motor vehicle accident

Water transport accident

Cutting and piercing instruments or objects

Accident caused by machinery

Explosive material

Assault

Not sure

Other

Display This Question:

If Q36 = Other

Q37 Please specify how your injury occurred.

____

Q38 Is there any other relevant information that you would like to mention? For example, the equipment you were wearing at the time or environmental conditions, like uneven terrain.

____

Start of Block: Not eligible

Display This:

If Q1 = No

And Q2 = No

And Q3 = No

Sorry, you are not eligible to partake in this study.
